# Extranodal NK/T-cell lymphoma in Tunisia: clinicopathological features, immunophenotype and EBV infection

**DOI:** 10.1186/s43046-019-0002-3

**Published:** 2019-10-22

**Authors:** Nabiha Missaoui, Sarra Mestiri, Aida Bouriga, Nihed Abdessayed, Mouna Belakhdher, Monia Ghammem, Mohamed Abdelkefi, Moncef Mokni, Sihem Hmissa

**Affiliations:** 1 Research Unit UR14ES17, Medicine Faculty of Sousse, University of Sousse, Sousse, Tunisia; 2 Pathology Department, Farhet Hached Hospital, Sousse, Tunisia; 3 Faculty of Sciences and Techniques of Sidi Bouzid, University of Kairouan, Kairouan, Tunisia; 4 Otorhinolaryngology Surgery Department, Farhet Hached Hospital, Sousse, Tunisia

**Keywords:** Extranodal NK/T-cell lymphoma, Nasal, EBV, Immunophenotype, Tunisia

## Abstract

**Background:**

Extranodal NK/T-cell lymphomas (ENKTL) are rare non-Hodgkin’s lymphomas with aggressive clinical behavior. ENKTL are frequently associated with the Epstein-Barr virus (EBV). Data on ENKTL in Africa and Arab world are extremely limited. The study investigated the clinicopathological characteristics, EBV infection, and immunophenotype of ENKTL in Tunisia. We conducted a retrospective study of ENKTL. Main clinicopathological features were reported. The expression of CD3, CD4, CD5, CD8, CD20, CD56, CD57, and Granzyme B were analyzed by immunohistochemistry. EBV infection was detected by IHC (LMP-1) and Epstein-Barr encoding region (EBER1/2) in situ hybridization.

**Results:**

A total of nine ENKTL were identified (mean age of 48 years and male-to-female ratio of 8:1). There were five nasal ENKTL, and the remaining four cases had extranasal involvement (palate, sub-mandibular gland, skin, and soft tissues of the ankle). The histopathology showed a lymphoid and pleomorphic proliferation characterized by images of angiocentrism. Strong and diffuse CD3 expression was observed in all cases. Tumor cells exhibited an expression of CD5 (two cases), CD8 (three cases), CD56 (six cases), CD57 (three cases), and Granzyme B (eight cases). All ENKTL cases were EBV-associated. Overall 5-year survival rate was 57%. Although six ENKTL were diagnosed at early clinical stages, the prognosis was unfavorable and associated with patient death in three cases.

**Conclusions:**

ENKTL are exceptional in Tunisia with unfavorable outcome. Histopathological diagnosis remains challenging in clinical practice. However, a careful histopathological examination combined with a correct interpretation of immunohistochemistry and in situ hybridization results refines the ENKTL diagnosis.

## Background

Extranodal NK/T-cell lymphoma (ENKTL) is a rare and aggressive non-Hodgkin’s lymphoma [1]. It was previously known as a lethal midline granuloma. ENKTL was recognized by the World Health Organization (WHO) in 2001 as a result of the histopathological progress associated with immunohistochemistry (IHC) in clinical practice [2]. ENKTL are reported mainly in East Asians and Native Americans in Mexico, Central America, and South America, accounting for 3–8% of non-Hodgkin’s lymphoma [3]. ENKTL characteristics in African and Arab patients are extremely limited [4–6].

Depending on the primary anatomical sites of the involvement, there were three forms of ENKTL, including nasal ENKTL, nasal-type ENKTL, and leukemic form of ENKTL, affecting the liver, spleen, bone marrow, and lymph nodes [7]. Typically, ENKTL causes destruction of the mid-face, palate, and orbital walls, but may also involve the skin, soft tissues, testes, gastrointestinal tract, bone marrow, and upper respiratory tract [8]. This lymphoma is characterized by vascular damage, necrosis, and cytotoxic phenotype. The ENKTL diagnosis is currently based on histopathology, IHC, and in situ hybridization (ISH). ENKTL represent a distinct clinicopathological entity etiologically associated with the Epstein-Barr virus (EBV) infection [9]. ENKTL evolution is spontaneously fatal, and the prognosis remains overall poor [10].

To further explore this rare non-Hodgkin’s lymphoma, we analyzed clinicopathological features, immunohistochemical profile, EBV infection, treatment modalities, and clinical outcomes of ENKTL in Tunisian patients.

## Methods

### Tissue samples

We carried out a retrospective study of ENKTL diagnosed at the Pathology Department and treated jointly in the Hematology, Medical Oncology, and Radiotherapy departments of the Farhet Hached University Hospital, Sousse (Tunisia), during 1997–2017. We excluded leukemic forms of NK/T lymphoma, which may present clinically with lymph node involvement. This study was approved by the local Human Ethics Committee at the Farhet Hached University Hospital of Sousse (Tunisia), and it conformed to the provisions of the Declaration of Helsinki.

All tissues had been routinely fixed in 10% buffered formalin and paraffin embedded. Hematoxylin- and eosin-stained sections of selected cases were reviewed by two pathologists (SM and MM) to confirm the diagnosis.

### Clinicopathological data

The collection of clinicopathological data was conducted using patient clinical records from Pathology, Hematology, Medical Oncology, and Radiotherapy departments of the Farhet Hached University Hospital, Sousse. Gender, age at diagnosis, cancer discovery circumstances, site of involvement, and Ann-Arbor classification were recorded. The modes of therapy used and the follow-up data were collected.

### Immunohistochemistry

Immunohistochemistry (IHC) was performed on sections of 4-μm thickness. After dewaxing and rehydration, the antigen unmasking was carried out in a citrate buffer at 95 °C for 40 min. The endogenous peroxidase activity was blocked by 3% hydrogen peroxide. Slides were then incubated with the primary antibody at room temperature (20–25 °C) for 30 min (Table 1). The revelation was made by the Envision+ Dual Link System HRP kite (Dako, code K4063). Finally, diaminobenzidine was used as the chromogen for the immunostaining and sections were counterstained with hematoxylin. Appropriate positive controls were performed according to the manufacturer’s instruction. Negative controls were obtained by substitution of the primary antibody by phosphate-buffered saline. IHC evaluation was independently performed by two pathologists (SM and MM).

**Table 1:** Immunohistochemistry conditions and evaluation

Antibody	Clone	Provenance	Dilution	Retrieval solution	Positive immunostaining
CD3	LN10	Novo Castra	1/50	Citrate buffer (10 mM, pH 9)	Intracytoplasmic staining
CD4	4B12	Novo Castra	1/20	Citrate buffer (10 mM, pH 9)	Cytoplasmic staining
CD5	4C7	Novo Castra	1/50	Citrate buffer (10 mM, pH 9)	Cytoplasmic staining
CD8	1A5	Novo Castra	1/30	Citrate buffer (10 mM, pH 9)	Cytoplasmic staining
CD20	L26	Dako	1/200	Citrate buffer (10 mM, pH 6)	Cytoplasmic staining
CD56 (N-CAM)	1B6	Novo Castra	1/50	Citrate buffer (10 mM, pH 6)	Cytoplasmic staining
CD57 (NK1)	NK1	Novo Castra	1/25	Citrate buffer (10 mM, pH 6)	Cytoplasmic staining
LMP-1	CS1-CS4	Dako	1/100	Citrate buffer (10 mM, pH 6)	Cytoplasmic staining
Granzyme B	GrB-7	Dako	1/25	Tris-EDTA buffer (pH 9)	Cytoplasmic staining

*LMP-1* latent membrane protein 1

### EBV infection

The analysis of EBV infection was carried out by the Epstein-Barr virus-encoded RNA (EBER) transcript ISH. Deparaffinized tissue sections were digested with proteinase K for 30 min at room temperature. Sections were then incubated overnight at 42 °C with fluorescein isothiocyanate-conjugated EBER PNA-probes (Y5200). After washing in Tris-buffered saline solution (pH 7.6), the slides were incubated with anti-FITC alkaline phosphatase-conjugated antibody followed by 4-nitroblue tetrazolium chloride/5-bromo-4-chloro-3-indolyl phosphate substrate combined with levamisole (K5201) according to the manufacturer’s protocol (DAKO, Code Y5200). Contamination with RNAses was avoided strictly. Cells exhibiting nuclear staining were scored positive. An EBV-positive lymphoma and EBV-negative lymphoid tissue were used separately as positive and negative controls, respectively.

## Results

### Epidemiological and clinicopathological findings

Table 2 summarizes the epidemiological and clinicopathological characteristics of ENKTL cases. During the study period, non-Hodgkin’s lymphomas accounted for 5.9% of all cancer cases diagnosed in our Pathology Department. Overall, nine ENKTL were identified, accounting for 0.78% of all non-Hodgkin’s lymphomas. Patient age ranged from 26 to 74 years with a mean age of 48 years. There was a male predominance with a male-to-female ratio of 8.1.

**Table 2:** Clinicopathological features of extranodal NK/T-cell lymphomas

Patient	Age	Gender	Site	Clinical stage	Treatment	Clinical outcome
1	69	Male	Left nasal cavity	IIE	CT (6× CHOP) RT (45 Gy)	Alive 10 years after the first diagnosis without tumor recurrence
2	30	Male	Right nasal cavity	IIE	CT (4× ACVBP)	Died 4 months after the first diagnosis
3	26	Male	Left nasal cavity	IIB	CT (4× ACVBP) RT (45 Gy)	Patient lost 1 year after treatment starting
4	39	Male	Left nasal cavity	IA	CT (4× ACVBP) RT (45 Gy)	Alive 6 years after the first diagnosis without tumor recurrence
5	54	Male	Palate	IE	CT (6× CHOP)	Died only 2 months after the first diagnosis
6	74	Male	Sub-mandibular lodge	IV	Surgery CT (6× CHOP) RT (45 Gy)	Alive 5 years after the first diagnosis without tumor recurrence
7	39	Female	Nasal cavities	IA	CT (4× ACVBP) RT (45 Gy)	Alive 7 years after the first diagnosis without tumor recurrence
8	50	Male	Soft tissue of ankle	Undetermined	Surgery	Patient lost after tumor resection
9	55	Male	Skin	IV	CT (6× SMILE) RT (62 Gy)	Died 2 months after CT end with meningeal relapse

*ACVBP* adriamycine, cyclophosphamide, vindesine,bleomycine, prednisone; *CHOP* cyclophosphamide, hydroxydaunorubicin, vincristine (oncovin), prednisone; *CT* chemotherapy; *RT* radiation therapy; *SMILE* dexamethasone, methotrexate, ifosfamide, l-asparginase, etoposide

Two patients presented a family history of endometrial cancer in the mother (patient 9) and a history of lymphoma in the sister and a bladder tumor in the father (patient 4). A non-neoplastic personal history was reported in two patients (patients 1 and 2). The diagnosis time ranged between 1 and 7 months with a mean of 4 months.

Considering the involvement site, there were five ENKTL located in the nasal cavity (cases 1, 2, 3, 4, and 9) and four extranasal ENKTL, interesting the soft palate (case 5), the sub-mandibular gland (cases 6), the skin (case 7), and the soft tissue (case 8).

The most common clinical signs of nasal ENKTL were unilateral or bilateral obstruction (five cases), rhinorrhea (three cases), and periorbital edema (two cases). For the extranasal ENKTL, the symptoms were essentially a swelling of the sub-mandibular gland, swelling of the ankle with functional impotence, and cutaneous tumor nodules of the knee and leg.

The rhinological examination revealed a necrotic and a superinfected budding ulcerous lesion of the nasal fossae (four patients). Inflammatory mucosa, covered with crusts and purulent secretions, was observed in two patients. A destruction of the nasal septum was observed in one patient. An impairment of the general condition was observed on initial examination in three patients.

According to the Ann-Arbor classification, the clinical stage was available for eight patients (Table 2). Six cases were diagnosed at localized clinical stages (I/II). The remaining two patients had advanced stage (III/IV) disease.

**Fig. 1:**
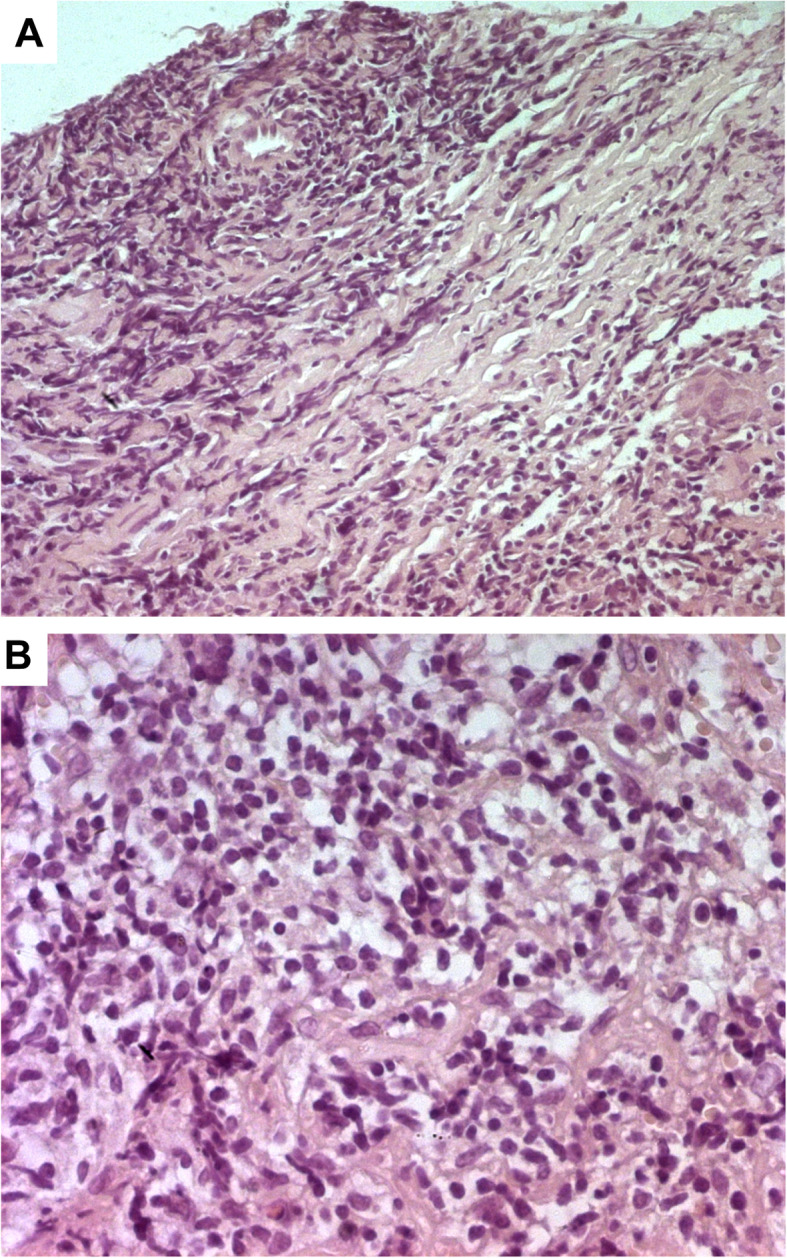
Histopathological features of extranodal NK/T-cell lymphoma. Diffuse malignant lymphoid proliferation made of pleomorphic tumoral cells with atypical and dense nuclei, and mitotic figures (**a** original magnification, × 200; **b** original magnification, × 400)

The histopathological aspect showed a malignant tumor proliferation of lymphoid and pleomorphic type, made of small, medium, and some large cells in eight cases (Fig. 1). Nuclei were atypical with irregular contours. In case 6, lymphoid cells were relatively monotonous of medium size with rounded or slightly irregular contours. Chromatin was variable in appearance: dense, finely granular, or marginated. The nucleolus was mostly small. The cytoplasm was abundant and pale. Mitoses were numerous in three cases. Moreover, there was a polymorphic inflammatory population, including lymphocytes, plasma cells, neutrophils, and eosinophils in four cases. In the other four cases, there was a population of small reaction lymphocytes. There were foci of necrosis with nuclear debris in cases 3, 4, 7, and 9. The lymphoid proliferation was also characterized by images of angiocentrism or angiotropism found in seven cases.

### IHC results

**Fig. 2:**
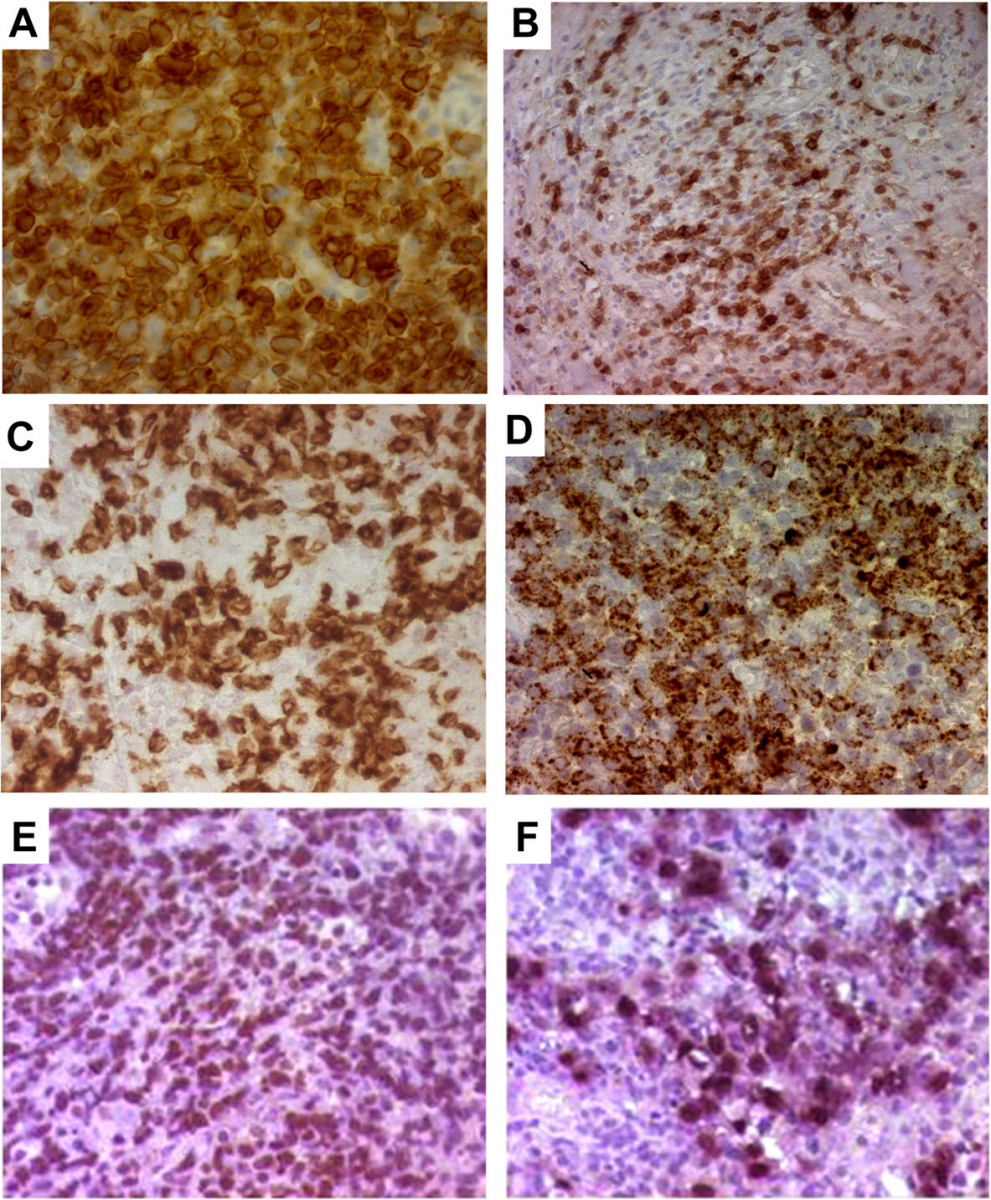
Immunohistochemical characteristics of extranodal NK/T-cell lymphoma. **a** CD3 expression (original magnification, × 400). **b** CD5 expression (original magnification, × 400). **c** Expression of CD56 (original magnification, × 400). **d** Granzyme B immunostaining of tumor cells (original magnification, × 400). **e** Positive LMP-1 immunostaining (original magnification, × 400). **f** EBER-positive tumor cells detected by in situ hybridization (original magnification, × 400)

The IHC findings are summarized in Table 3. Tumor cells exhibited an intense and diffuse cytoplasmic CD3 expression in all analyzed cases (Fig. 2a). The expression of CD5 and CD8 was detected in two cases and three cases, respectively (Fig. 2b). No expression of CD4 and CD20 was found. CD57 was expressed in three cases. Tumor cells expressed CD56 in six cases and Granzyme B in eight cases (Fig. 2c, d). Co-expression of CD56 and Granzyme B was found in five cases.

**Table 3:** Immunohistochemical and EBER in situ hybridization results

	Patient 1	Patient 2	Patient 3	Patient 4	Patient 5	Patient 6	Patient 7	Patient 8	Patient 9	Total
CD3	+	+	+	+	+	++	+	++	+	9 (100%)
CD4	–	–	–	–	–	–	–	–	–	–
CD5	–	–	+	–	–	+	–	–	–	3 (33.3%)
CD8	–	+	+	+	–	–	–	–	–	3 (33.3%)
CD20	–	–	–	–	–	–	–	–	–	–
CD56 (N-CAM)	+	–	+	+	–	+	–	++	+	6 (66.7%)
CD57 (NK1)	–	+	–	+	–	+	–	–	–	3 (33.3%)
Granzyme B	+	+	+	+	+	–	+	+	+	7 (77.8%)
LMP-1	–	+	+	–	+	+	–	–	–	4 (44.4%)
EBER ISH	+	+	+	+	+	+	+	+	+	9 (100%)

+ positive staining, ++ strong expression, − negative staining, *EBER* Epstein-Barr encoding region, *ISH* in situ hybridization, *LMP-1* latent membrane protein 1

### EBV infection

Overall, the nine ENKTL were EBV-associated. EBV latency protein LMP-1 was expressed in four cases (cases 2, 3, 5, and 6) (Fig. 2e). Using ISH, EBER probes demonstrated the presence of viral genomes in all cases (Fig. 2f). EBV was detected simultaneously by both IHC and ISH techniques in four cases (Table 3).

### Treatment modalities

All patients received poly-chemotherapy except for one patient who was lost to the follow-up after surgical treatment (patient 8). A chemo-radiotherapy combination was indicated in five patients (cases 1, 3, 4, 7, and 9). Only patient 6 received a combination chemotherapy-surgery (Table 2).

The protocol of neo-adjuvant chemotherapy included CHOP (cyclophosphamide, hydroxydaunorubicin, vincristine (oncovin), prednisone) for two patients (cases 1 and 6), ACVBP (adriamycine, cyclophosphamide, vindesine, bleomycine, prednisone) for three patients (cases 3, 4, and 7), and SMILE (dexamethasone, methotrexate, ifosfamide, l-asparginase, etoposide) for patient 9. Chemotherapy alone was indicated in patients 2 and 5, using ACVBP and CHOP protocols, respectively.

Only five patients received adjuvant external radiotherapy (patients 1, 3, 4, 7, and 9). The radiotherapy was delivered at a rate of 2 Gy by session and five sessions by week during 4 to 7 weeks for a total dose between 40 and 68 Gy. No indication of exclusive radiotherapy as initial ENKTL treatment was reported.

Surgery was proposed in two patients. For the patient diagnosed with ENKTL in sub-mandibular, the intervention consisted of a large excision of the sub-mandibular mass with cervical dissection and mass biopsy (patient 6). For the patient with ENKTL localized in soft tissue, the treatment involved excision of the tumor tissue (patient 8).

### Clinical outcomes

The follow-up period ranged from 2 months to 10 years, with a mean of 2 years and 3 months. Overall 5-year survival rate was 57%. A favorable prognosis was reported in four cases, since a complete response was obtained in these patients (cases 1, 4, 6, and 7): three of which were diagnosed at an early clinical stage (Table 2). The evolution was unfavorable in three cases and uncertain in two patients (cases 3 and 8).

## Discussion

In this study, we investigated particularities of nasal and nasal-type ENKTL diagnosed in Tunisia, a non-endemic region, during a 15-year period. ENKTL is a rare lymphoma subtype of peripheral T/NK cell lymphoma with a variable incidence according to geographical region. ENKTL were more frequently reported in Asia and Latin America [3]. Indeed, ENKTL accounts for 12.9–25% of all non-Hodgkin’s lymphomas in China, Thailand, and Singapore [3, 11]. Europe and North America were less affected [12]. In Tunisia, ENKTL remains an exceptional tumor since only nine cases were reported during the study period.

ENKTL mainly occur in the fourth and fifth decades, with a mean age of 50 years [13]. In our series, the patient mean age was 48 years. In Asian series, the patients were younger than in the Western series [14]. Pediatric ENKTL remains exceptional [15, 16]. Although few series found a female preponderance, male predominance has been reported by several series with a male-to-female ratio ranging from 2 to 4.5 [1, 13]. Interestingly, herein, we observed a large male predominance.

The nasal ENKTL are localized in the nasal cavities, paranasal sinuses as well as the cavum, tonsils, hypopharynx, and larynx [17]. Nasal ENKTL are characterized by progressive-stage ulceronecrotic lesions in the medial structures of the face, preferentially in the nasal fossae and the sinuses [17]. Nasal ENKTL can also develop in the Waldeyer’s ring, oral cavity, and hypopharynx. The nasopharynx has been rarely involved [18]. In our series, NKTL were diagnosed in the nasal cavity (five cases), the soft palate, and the sub-mandibular gland. In addition, nasal-type ENKTL may involve the skin, soft tissues, testes, gastrointestinal tract, bone marrow, liver, and upper respiratory tract [17]. However, tracheal and laryngeal localizations were rare [19]. Herein, we reported two nasal-type ENKTL involving the skin and the soft tissue.

According to the Ann-Arbor classification, early clinical stage ENKTL were the most commonly diagnosed forms at the time of diagnosis with approximately two thirds of patients having localized disease in the nasal cavity or adjacent sites [14, 17]. However, other reports described either only early clinical stages [13] or mainly metastatic stages [18]. In our series, ENKTL were diagnosed predominantly in early stage (six cases), the remaining two cases were diagnosed at disseminating stages.

The histopathological diagnosis is particularly difficult, because of the frequent entanglement of infected and necrotic-inflammatory phenomena with lymphoid proliferation. Hence, the biopsy must be performed at a distance from the necrosis. T/NK cell lymphoma showed polymorphic lymphoid proliferation, consisting of small, medium, and large cells, with atypical irregularly contoured nuclei [18]. The tumor proliferation is most often associated with a polymorphic inflammatory infiltrate made of lymphocytes, eosinophilic polynuclear cells, plasma cells, and histiocytes. Ischemic necrosis was frequently observed and associated with lesions of angiocentrism [17].

The differential diagnoses of T/NK lymphoma are mainly the Wegener’s disease and bacterial and fungal infections. Nevertheless, IHC remains essential to establish the diagnosis [17]. As ENKTL develops from cytotoxic cells, mainly NK cells and more rarely T cells, they exhibit intracytoplasmic CD3 expression as well as CD2 and CD56 expression [17, 18, 20]. However, less than 5% of T lymphocytes and a small proportion of NOS lymphomas can express CD56 [18]. Although the significance of the expression of this adhesion molecule remains unknown, CD56-positive lymphomas are characterized by an extranodal invasion, a high aggressivity, and a poor prognosis [21]. Herein, six ENKTL expressed CD56.

NK/T lymphomas are most often CD4−/CD8−, more rarely CD4−/CD8+ [17]. In our study, CD4 was negative in all cases and CD8 was expressed in three ENKTL. CD57 can be expressed [22]. NK/T lymphomas usually have an activated cytotoxic phenotype, expressing the cytotoxic granule-associated proteins, such as T1A, Granzyme B, and Perforine [17]. In the current study, the expression of Granzyme B was detected in eight ENKTL. Moreover, there is a strong correlation between the expression of CD56 and that of Granzyme B.

During the last decade, EBV detection by ISH and IHC demonstrated the association of ENKTL with EBV infection, regardless of ethnic and geographical distribution [8, 23]. Using novel techniques, such as DNA microarrays, array comparative genomic hybridization, and “next-generation” sequencing, the oncogenesis of EBV-associated NK/T lymphomas are now being clarified, although the exact molecular mechanisms remain unresolved [23–25]. Overall, among immunocompetent patients, EBV-infected cells are most frequently reported in NK/T lymphomas and angioimmunoblastic T lymphomas than in B lymphomas, suggesting an important role of this virus in the pathogenesis of these lymphomas [23, 24]. Interestingly, herein, all ENKTL were EBER-positive similar to Northern Chinese patients [18]. Takada et al. showed recently that EBV induces NF-κB-mediated survival signals in T and NK cells, contributing to the lymphomagenesis of these cells [26].

The optimal treatment of ENKTL remains poorly established due to the rarity of this lymphoma and the small sample size of the randomized controlled trials. Studies have clearly shown that the clinical response to chemotherapy in localized forms is unfavorable, since a complete response is observed in only 40 to 60% of patients [1]. In our study, patient 2 died due to bone marrow failure 4 months after diagnosis and patient 8 had bone marrow failure and died after meningeal relapse. Radiation therapy followed by or in combination with chemotherapy is the best initial treatment since it improves the local control of the disease and reduces the local and systemic recurrence [17, 18, 21]. However, it has not been well-established whether this association can provide a benefit for survival. Most studies have shown that conventional chemotherapy followed by radiotherapy seems to be ineffective for the majority of patients [1, 23]. As a result, it has been suggested that current chemotherapy protocols should be reserved for the control of micro-metastases following radiotherapy [1]. Herein, the radio-chemotherapy combination was indicated in five patients, and the median survival of this group of patients was longer than that of the exclusive multi-chemotherapy group (5 years 8 months versus 1 month 15 days).

Since nasal-type ENKTL is an aggressive and persistent disease, conventional chemotherapy is insufficient for the eradication of this lymphoma. Some studies have suggested that high-dose chemotherapy, supported by stem cell transplantation, with a combination of an l-asparginase-based regimen can significantly improve the “anti-tumor effect” and eventually overcome drug resistance [17, 27]. In a prospective study of 29 newly diagnosed nasal ENKTL, after two to three courses of the SMILE protocol, 24 cases showed a response rate of 86% with a complete response obtained in 69% of patients [27]. l-Asparginase as a single agent is effective in treating recurrent or treatment-resistant ENKTL [27, 28]. Nevertheless, in our study, only one patient received SMILE protocol and died, in a context of meningeal relapse, complicated of febrile pancytopenia. Although this worse outcome, SMILE regimen remains the current standard of care in many countries for ENKTL patients of diverse age groups and performance status, with newly diagnosed or relapsed/refractory tumors and in early to advanced stages [27].

Post-treatment surveillance of ENKTL patients should be clinical, biological, and radiological. The quantification of the circulating copy rate of the EBV DNA by polymerase chain reaction technique is a predictive parameter of relapse or recurrence of this lymphoma [17, 29]. The prognosis overall remains poor with a median survival of only 13 months [7, 13]. The estimated 5-year overall survival is between 40 and 50% [12]. Survival is heavily dependent on stage at diagnosis. Patients with an early clinical stage have higher 5-year survival rates regardless of the type of treatment received. This rate is even lower for advanced stages not exceeding 10% [12]. In our study, the evolution was favorable in only four cases in which a complete response was obtained (patients 1, 4, 6, and 7) of which three cases were classified at early clinical stages.

Similar to other EBV-associated cancer, novel immunotherapy options targeting LMP1 protein may improve clinical outcomes for ENKTL patient [30]. In two clinical trials of EBV-cytotoxic T lymphocytes as an adjuvant therapy, durable responses were observed [17].

## Conclusion

The current study confirms that ENKTL deserves to be distinguished from other T lymphomas, since this lymphoma tends to occur in young people, to spread early, to have a pejorative evolution, and to be strongly associated with EBV infection. Although ENKTL are exceptional in Tunisia, clinicopathological features, EBV status, and immunophenotype were similar to those described in previous reports from endemic regions. The histopathological diagnosis remains challenging in clinical practice. However, careful examination with a correct interpretation of the IHC and ISH results generally improve ENKTL diagnosis. ENKTL remain aggressive disorders with unfavorable outcome and frequent relapse after complete remission, in which targeted treatments are still in a preliminary phase. More multicenter studies, using much larger series, would be necessary to evaluate these therapeutic strategies.

## Data Availability

Not applicable.

## Abbreviations

EBER: Epstein-Barr encoding region
EBV: Epstein-Barr virus
ENKTL: Extranodal NK/T-cell lymphomas
IHC: Immunohistochemistry
ISH: In situ hybridization
LMP-1: Latent membrane protein 1
